# The role of N-acetylcysteine and glutathione in the management of Parkinson’s disease: a systematic review of oxidative biomarkers and clinical outcomes

**DOI:** 10.1007/s00726-026-03513-5

**Published:** 2026-03-24

**Authors:** Imran Mohammed, Yasimin Nankya, U. Teng Hong, Amy Kan, Alya Masoud Abdelhafid, Gladys O. Latunde-Dada

**Affiliations:** 1 GKT School of Medical Education, Faculty of Life Sciences & Medicine, King’s College London, London, UK; 2 Department of Nutritional Sciences, School of Life Course and Population Sciences, Faculty of Life Sciences & Medicine, King’s College London, London, UK; 3 Department of Neuromuscular Diseases and UCL Queen Square Motor Neuron Disease Centre, UCL Queen Square Institute of Neurology, University College London, London, WC1N 3BG UK

**Keywords:** N-acetylcysteine, Glutathione, Oxidative stress, Parkinson’s disease, Neuroprotection, Dopamine transporter

## Abstract

Parkinson’s Disease (PD) is a progressive neurodegenerative disorder characterised by the loss of dopaminergic neurons, leading to both motor and non-motor symptoms. Oxidative stress is a significant contributor to the pathophysiology of PD, and glutathione (GSH) depletion contributes to neuronal damage. N-acetylcysteine (NAC), a bioavailable cysteine donor, can support endogenous GSH synthesis and may also exert antioxidant effects independent of GSH replenishment. NAC and GSH are proposed neuroprotective interventions due to their antioxidant properties. This systematic review evaluated the effects of NAC and GSH on oxidative stress and PD symptoms, comparing them with healthy controls or a placebo. A systematic search was conducted in Cochrane Library, PubMed, Web of Science, Ovid (Embase and MEDLINE), Scopus, and ProQuest for studies published between January 2003 and December 2024, including randomised controlled trials (RCTs) and non-randomised studies. Two reviewers assessed the study quality and extracted data. The primary outcome was the change in motor and non-motor symptoms as measured by the Unified Parkinson’s Disease Rating Scale (UPDRS) and were interpreted using minimal clinically important difference (MCID) thresholds. Secondary outcomes included biochemical redox markers such as blood GSH, cerebrospinal fluid (CSF) GSH, GSSG, and GSH/GSSG ratio and imaging-based functional outcomes, particularly DAT binding assessed by DaTscan SPECT. The GSH/GSSG ratio reflects redox status (reduced vs. oxidised glutathione), CSF outcomes primarily reported NAC concentrations, and brain GSH was quantified using magnetic resonance spectroscopy (MRS). Exclusion criteria included studies on conditions other than PD, those that did not use NAC or GSH as the primary intervention, and those without a comparator group. Nine studies, conducted between 2009 and 2019, met the inclusion criteria and involved 196 participants. NAC improved both motor and non-motor symptoms and significantly increased GSH/GSSG ratios, GSH levels in the CSF, and DAT binding. In contrast, intranasal GSH showed only modest increases in brain levels without significant improvements in symptoms or oxidative stress markers. The studies had limitations, including small sample sizes, short intervention durations, and inconsistencies in dosage and administration routes. These factors constrain the strength of the conclusions, and evidence for both NAC and GSH remains preliminary. Furthermore, while NAC shows promise as a neuroprotective intervention, findings for GSH are inconclusive. More large-scale, long-term randomised controlled trials are needed to validate these results and explore NAC and GSH’s long-term therapeutic potential in managing PD.

**Supplementary Information:**

The online version contains supplementary material available at 10.1007/s00726-026-03513-5.

## Introduction

Parkinson’s Disease (PD) is a progressive neurodegenerative disorder caused by the degeneration of dopaminergic neurons in the substantia nigra, a region of the brain responsible for controlling movement. It is the second most common neurodegenerative disorder after Alzheimer’s Disease. Ageing is the most significant risk factor of PD, with most cases occurring in patients over 60 years old (Lees et al. 2009). The exact aetiology of PD is not fully understood, although it is believed to result from a combination of genetic and environmental factors. Mutations in genes such as SNCA, LRRK2 and PARK2 have been linked to familial and sporadic forms of the disease (Deng et al. 2018). Environmental exposures, including pesticides, herbicides, and heavy metals, have also been reported as potential contributors (Ball et al. 2019). Symptoms of PD can be categorised into motor symptoms that include bradykinesia, rigidity, tremor, and postural instability, which result from the depletion of dopamine in the basal ganglia. Non-motor symptoms include cognitive impairment, mood disturbances, sleep disorders, and autonomic dysfunction (Gokcal et al. 2017). There are several treatment options available, primarily aimed at restoring dopamine levels in the brain (Kouli et al. 2018). However, there is currently no cure for the condition, and due to the variability in how the disease presents itself, some symptoms may not respond effectively to these treatments. Available treatments such as levodopa, dopamine agonists and monoamine oxidase B inhibitors offer only symptomatic relief however, they do not treat the underlying neurodegeneration, emphasising the need for neuroprotective management. Consequently, further research is necessary to explore therapies that can slow down neurodegeneration and the progression of the disease.

Oxidative stress is involved in the pathophysiology of PD as excessive production of reactive oxygen species (ROS) contributes to dopaminergic neuronal damage, which is susceptible to oxidative injury due to high metabolic activity and dopamine metabolism. Disruption of the antioxidant pathway or depletion of glutathione (GSH) in the brain may contribute to the development of PD (Dias et al. 2013), indicating the potential of GSH as a treatment option. N-acetylcysteine (NAC) and GSH antioxidants have demonstrated potential neuroprotective effects in preclinical studies of augmenting central nervous system (CNS) GSH concentrations. NAC is a thiol-containing compound that acts as a cysteine donor which can cross the blood-brain barrier (BBB) and replenish intracellular GSH levels thereby supporting endogenous GSH synthesis, while GSH interventions can restore antioxidant capacity and reduce oxidative stress. NAC can also exert antioxidant and anti-inflammatory effects independent of GSH replenishment (Mischley et al. 2016).

Several natural compounds have been proposed as interventions for PD including coenzyme Q10, curcumin, resveratrol and omega-3 fatty acids, however these agents have shown limited clinical efficacy, often failing to produce measurable changes in Unified Parkinson’s Disease Rating Scale (UPDRS) outcomes or biomarkers of oxidative stress. (Bhusal et al. 2023). NAC and GSH were prioritised because they target GSH biology more directly than these alternatives; GSH is the primary endogenous antioxidant depleted in PD and NAC promote glutathione homeostasis indirectly by providing cysteine for GSH synthesis. Earlier narrative reviews of NAC and GSH report limited early phase data without directly comparing the two antioxidants in terms of motor and non-motor outcome measures (Mischley et al. 2016). Despite preliminary findings, the clinical efficacy and safety of these interventions in PD in these earlier studies remain inconclusive, with variability in reported outcomes. Therefore, the aim of this study is to address this gap by incorporating formal risk-of-bias assessment, to evaluate the relevance of PD using UPDRS minimal clinically important differences (MCIDs). This involves juxtaposing NAC and GSH across clinical outcomes such as motor and non-motor symptoms of PD using UDPRS scores. Moreover, mechanistic outcomes such as oxidative stress markers, GSH/GSSG ratio and DAT binding were compared to healthy controls, placebo or comparison to baseline.

## Methods

The study followed the Preferred Reporting Items for Systematic Reviews and Meta-Analyses (PRISMA) guidelines (Page et al. 2021). PROSPERO Registration, CRD42023446947.

### Eligibility criteria

The PICOS criteria for inclusion and exclusion are shown in Table 1. The inclusion criteria consisted of observational studies and randomised control trials (RCT) involving human participants diagnosed with PD according to established diagnostic criteria e.g., UK Parkinson’s disease Society Brain Bank Criteria (Munhoz et al. 2024) to maximise diagnostic specificity and reduce misclassification with other Parkinsonian disorders. Only studies involving adults (≥ 18 years) were included to avoid the heterogeneity introduced by juvenile or atypical Parkinsonian syndromes, which may differ in aetiology, progression, and treatment response. Both RCTs and non-randomised experimental studies were included to obtain the full scope of available human evidence on NAC and GSH in PD. Non-randomised studies were included as they primarily address pharmacokinetic, neuroimaging, and mechanistic endpoints, such as central nervous system bioavailability and glutathione redox status, which are often not adequately evaluated in the RCT studies. Eligible studies evaluated NAC or GSH administered as an intervention in adults with PD; observational studies measuring endogenous GSH without NAC or GSH administration were excluded.

Studies including healthy individuals were not eligible unless they were incorporated as a comparator or control group alongside participants with PD. Non-English studies were excluded as validated translations of PD rating scales and risk-of-bias tools were not consistently available. Furthermore, unpublished studies often lacked sufficient methodological detail for quality appraisal, outcome data, and long-term follow-up, and the absence of peer review made it difficult to appraise internal validity in a standardised way. These exclusions were applied to maintain data reliability, although the potential for language and publication bias is acknowledged.

### Search strategy and study selection

Two independent reviewers conducted a systematic literature search using the Cochrane Library, PubMed, Web of Science, Ovid (Embase and MEDLINE), Scopus, and ProQuest for studies published between January 2003 and December 2024, with the final search being completed on 31st December 2024. This review summarises the most recent evidence and interventions. The following MESH headings, Embase thesaurus and keywords were searched: Parkinson’s disease or Parkinsonism or Parkinsonian symptoms and N-acetylcysteine or NAC or N-acetyl-L-cysteine or N Acetyl Lcysteine or N Acetylcysteine or acetylcysteine or glutathione or GSH or GSSG. A specialist librarian was recruited to validate the search string derivation. Screening from the title, abstracts and data extraction from the full texts against eligibility criteria were also carried out by two independent reviewers. If required, a third author resolved any disagreement about the inclusion or exclusion of an article. Duplicates were removed using EndNote.

### Primary and secondary outcomes

The primary outcome measure were changes in motor and non-motor symptom severity assessed through standardised rating scales, such as the UPDRS or the Movement Disorder Society-Unified Parkinson’s Disease Rating Scale (MDS-UPDRS), including total scores and motor (Parts II–III) and non-motor (Part I) subscales. (Hendricks and Khasawneh 2021). These scores were interpereted using MCIDs where applicable. Additional clinical measures such as Montreal Cognitive Assessment (MoCA), Activities of Daily Living (ADL) using UDPRS Part II motor scores, Parkinson’s Disease Questionnaire-39 (PDQ-39) and Patient-Reported Outcomes in Parkinson’s Disease (PRO-PD) were treated as supportive. Secondary outcomes were categorised into: (i) blood or CSF biochemical redox markers such as GSH, GSSG, GSH/GSSG ratio, cysteine and 4-hydroxynonenal (4-HNE), (ii) brain biochemical imaging markers such as H-MRS-derived brain GSH, and (iii) imaging-based functional outcomes, inlcuding DAT binding assessed by DaTscan SPECT, which were interpreted as supportive mechanistic evidence rather than primary indicators of clinical efficacy.

### Data extraction

Data was extracted on various aspects, including: study title; author(s) and year of publication; country in which the study was conducted; start and end date; sample size; participant characteristics (mean age); study design; number of participants completing the study and dropout rates (including reasons for dropout); details of the interventions (N-acetylcysteine and GSH dosage, duration, route of administration); method of participant recruitment; inclusion and exclusion criteria; characteristics of the control group; primary and secondary study aims; variables measured at baseline and post-intervention; change in PD motor and non-motor symptoms and blood/brain GSH concentrations; reported adverse effects and the authors’ conclusions regarding the efficacy, safety, and neuroprotective potential of the intervention. The full data extraction process was performed independently by two reviewers using a standardised, piloted template. To minimise errors, formalized double data entry was implemented followed by cross-validation in which both datasets were compared line-by-line to identify and resolve discrepancies. Any further discrepancies were resolved through third-reviewer adjudication. Data that was not found in the studies was coded as missing and not included in the final data extraction. Non-standardised outcome measures such as differing UPDRS subscales and biomarker units were synthesised descriptively to ensure consistency across studies.

### Assessment of risk of bias and effect sizes

The Cochrane Risk of Bias tool (Sterne et al. 2019) was applied to assess the risk of bias in RCT studies, and the Newcastle-Ottawa Scale (Wells et al. 2021) was used for cohort and case-control studies. This assessment evaluated the following types of bias, selection bias, performance bias, detection bias, attrition bias and reporting bias. Domains for RCT studies were rated as low risk (+) when methods were clearly adequate, high risk (–) when methods were absent or likely to introduce bias, and unclear risk (?) when insufficient detail was reported (Table 5). Non-randomised studies were rated as ≥ 7 stars classified as low risk, 3–6 stars as moderate risk, and 0–2 stars as high risk of bias, based on selection, comparability, and outcome assessment domains (Table 6). Two independent reviewers conducted the risk of bias assessment in a prespecified sequence, evaluating selection, performance, detection, attrition, and reporting bias domains in order. Discrepancies between reviewers were identified through a structured comparison process and resolved through consensus and any further disagreements were resolved through evaluation with a third reviewer. Final ratings for each study reflected unanimous agreement. The effect sizes for the included studies were calculated using Cohen’s d, with values interpreted as small (0.2–0.5), medium (0.5–0.8), and large (> 0.8). For each study, Cohen’s d was derived from the difference between pre- and post-intervention mean scores, divided by the pooled standard deviation (SD) of the pre- and post-intervention values. The pooled SD was calculated using standard formulas based on the reported SDs and sample sizes. When both pre- and post-intervention means and SDs were available, change scores were used in preference to post-treatment values alone. Studies that did not report sufficient variability data were excluded from effect size calculation.

### Data synthesis

The studies varied in terms intervention type, dosage regimens, duration of treatment, study design, and participant characteristics such as disease stage and concurrent medication hence given the substantial heterogeneity, a quantitative meta-analysis was not performed. These factors precluded statistical pooling of effect sizes or reliable subgroup comparisons. Therefore, findings were synthesised narratively in accordance with PRISMA guidelines (Page et al. 2021) for non-combinable data. Quantitative outcomes such as changes in UPDRS scores, GSH levels, and DAT binding were summarised descriptively, while qualitative patterns in clinical response and biomarker trends were analysed thematically to highlight consistency and directionality across studies.

**Table 1:** PICOS criteria for inclusion and exclusion of studies

PICOS criteria	Inclusion	Exclusion
Population	Humans diagnosed with PD based on recognised diagnostic criteria Studies involving adults (≥ 18 years)	Studies on conditions other than PD Studies involving healthy individuals or other unrelated neurological disorders
Intervention/exposure	Studies investigating NAC or GSH as primary interventions NAC or GSH used alone or in combination with other treatments for PD Any dosage, route of administration, or treatment duration	Studies where NAC or GSH is not the primary intervention Studies focusing solely on NAC or GSH for conditions other than PD
Comparator	Placebo, no treatment or comparison with baseline or day 0 of intervention if applicable. Comparators such as other neuroprotective agents, antioxidants, or interventions targeting oxidative stress.	Studies without a comparator group Comparisons involving irrelevant or undefined interventions
Outcome	Primary outcomes: Change in PD motor or non-motor symptoms Secondary outcomes: Change in internal GSH and NAC levels and dopamine transporter (DAT) binding	
Study design	Randomised controlled trials (RCTs), cohort studies and case-control studies investigating NAC or GSH in PD	Non-original studies, such as reviews, meta-analyses, editorials, conference abstracts, and commentaries. Studies without full text or unpublished studies Non-English language studies

## Results

### Study selection

**Fig. 1:**
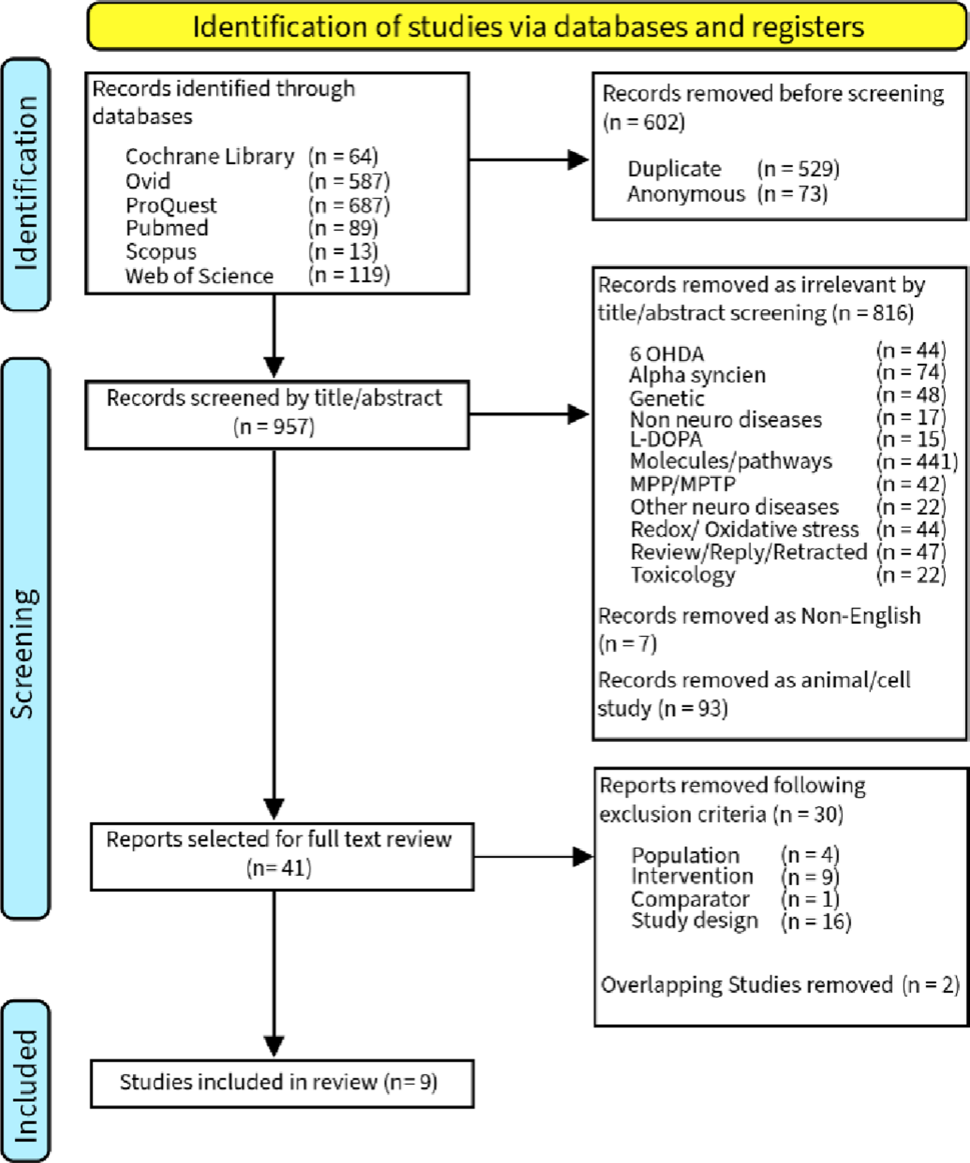
PRISMA flow diagram of the selection process. PRISMA flow diagram showing identification, screening, eligibility assessment, and inclusion of studies, with reasons for exclusion

The study selection process involved identification of studies, screening and inclusion using the PICOS criteria. Figure 1 shows the PRISMA flow diagram summary of the study selection process. A total of 1559 studies were identified by two independent researchers using Cochrane Library, PubMed, Web of Science, Ovid (Embase and MEDLINE), Scopus, and ProQuest (2001–2024). 529 duplicates were removed using Endnote; a further 73 studies were removed due to author details not being found, leaving 957 unique studies for screening. After title and abstract screening, a total of 916 studies were removed as Non English (*n* = 7), animal and cell studies (*n* = 93) and studies grouped as irrelevant; including studies focusing on 6-OHDA (*n* = 44), studies focusing on alpha synuclien (*n* = 74), studies focusing on genetic (*n* = 48), studies on non-neurological diseases (*n* = 17), studies focusing on effect of Levodopa (*n* = 15), studies focusing on novel molecules, food extract and their pathways in effecting neuro health (*n* = 441), studies focusing on MPP/MPTP (*n* = 42), studies conducted on other neurological disease (*n* = 22), studies focusing on redox reaction of GSH (*n* = 44), studies on toxicology (*n* = 22) and studies that are review, reply or retracted (*n* = 47).

A total of 41 studies were assessed in full-text review, with 30 studies excluded for the following reasons: 4 studies examined on neurological diseases other than PD such as Huntington’s disease and neurological diseases as a group; 9 studies where NAC/GSH is not the primary intervention; ;1 report has no comparator as it is a case report; 16 studies were review, meta-analysis and commentary and 2 studies were identified as overlapping studies. Ultimately, 9 studies met the inclusion criteria and were included in the final review.

### Characteristics of selected studies

#### Participants

The studies involved patients diagnosed with PD, with ages ranging from 18 to 84 years. All the studies documented ages across a range; apart from Mischley et al. (2017) which provided a mean age of 60.9 years for participants (Table 2). Sample population sizes varied across the 9 studies, ranging between 9 and 45 participants. Two papers also included healthy controls (Holmay et al. 2013; Coles et al. 2018) for baseline analysis of brain and blood GSH levels. Exclusion rates were kept to a minimum, with 13 patients across studies (13/196; 6.6%) withdrawing from clinical trials for the following reasons: adverse reactions, logistical barriers such as not being able to attend trials, or discomfort related to interventions such as lumbar punctures or sinus irritation (Table 3). These withdrawals are distinct from exclusions during literature screening. All the studies used were conducted in the United States.

#### Study design and methods used

Included studies comprised of non-randomised experimental studies (Holmay et al. 2013; Katz et al. 2015; Mischley et al. 2016; Coles et al. 2018) and randomised controlled trials (RCTs) (Monti et al. 2016, 2019; Mischley et al. 2015, 2017; Hauser et al. 2009). Non-randomised studies primarily focused on assessing oxidative stress indirectly using biochemical markers and RCTs examined clinical outcomes and safety. Key methods to measure outcomes included brain imaging using H-MRS to measure CNS GSH levels (Holmay et al.,2013, Mischley et al. 2016), radioligand SPECT imaging DaTscan as an indirect measure of DAT binding (Monti et al. 2016, 2019), and comprehensive blood analysis for oxidative stress markers and liver/renal function tests (Coles et al. 2018; Mischley et al. 2017).

#### Variables measured

Baseline assessments included demographic variables (e.g., age, gender, ethnicity), disease-specific measures such as Hoehn and Yahr staging, which is a clinical scale of PD severity and motor symptom progression, disease duration, and medication use. Clinical assessments included UPDRS and, where reported, MoCA and validated patient-reported outcome measures such PDQ-39 and PRO-PD. Post-intervention variables were targeted toward the primary outcomes of interest: motor symptom changes (UPDRS scores), CNS and blood GSH levels (using H-MRS or blood sampling), CSF GSH levels and dopamine function (DAT binding from DaTscan). Safety outcomes, including adverse events, vital signs, and electrocardiogram (ECG) changes, were also monitored.

**Table 2:** Study characteristics of participants and outcomes

References	Year	Country	Age (years)	Study design	Primary aim of study	Other aims of study
Holmay et al.	2013	United States	18–64	Non-randomised experimental study	Determine if NAC is able to alter peripheral and central redox capacity in patients with PD or Gaucher disease	
Katz et al.	2015	United States	48–81	Non-randomised experimental study	Determine whether biologically relevant levels of NAC could be achieved in human CSF after oral administration	
Mischley et al.	2016	United States	44–83	Non-randomised experimental study	Evaluate whether GSH is capable of increasing GSH concentrations in the CNS, measured by H-MRS	
Coles et al.	2018	United States	> 18	Non-randomised experimental study	Evaluate the effect of NAC on blood and brain GSH following repeated oral dosing	Measure tolerability and clinical outcomes using UPDRS Evaluate NAC’s effect on other systemic measures of oxidative stress and 4-HNE Determine if brain GSH concentrations measured using 3T MRS were comparable to those obtained at 7T
Monti et al.	2016	United States	40–80	Randomised controlled trial	Whether administration of NAC over three months would result in improved dopamine function as reflected by increased DAT binding on DaTscan and UPDRS scores	Confirm that NAC would have a neuroprotective effect in cultures of hESC-derived mDA neurons treated with rotenone (cell line study - not related to our review)
Monti et al.	2019	United States	40–80	Randomised controlled trial	Provide expanded data regarding effect of NAC administration on DAT binding and symptoms in patients with PD	Assess whether specific factors such as age, gender, disease duration and severity or medication use might interact with changes observed with NAC
Mischley et al.	2015	United States	> 21	Randomised controlled trial	Evaluate safety and tolerability of intracellular GSH in a cohort of individuals with PD	
Mischley et al.	2017	United States	> 21	Randomised controlled trial	Determine whether UPDRS total and motor improvements are seen (as in phase I/IIa), and evaluate whether phase III study is warranted	Describe systemic absorption characteristics of intracellular GSH and tolerability of intracellular GSH in patients with PD
Hauser et al.	2009	United States	43–84	Randomised controlled trial	Evaluate the safety, tolerability and preliminary efficacy of IV GSH in patients with PD	

**Table 3:** Characteristics of study design variables and patient sample

References	Start date	End date	Sample at baseline	Sample completing study	Number excluded and reasons	Variables measured at baseline	Variables measured post-intervention
Holmay et al. (2013)	Jul-11	Dec-12	9	9	0	Age, ethnicity, gender, disease duration, weight, smoking status, use of alcohol and illicit drugs, UPDRS (I–III), Hoehn & Yahr staging, Montreal Cognitive Assessment Score, medications, supplements and vital signs. Following collection of blood sample, subject was placed in MR scanner to determine brain GSH concentration	Subject placed back in MR scanner ~ 30 min after start of infusion, brain GSH and blood samples taken every 15 min until 1 h after end of infusion
Katz et al. (2015)	Nov-14	Feb-15	13	12	1 - post-lumbar puncture headache	Lumbar punctures, CSF analysed for cell count, protein, glucose, NAC, cysteine and GSH levels	CSF concentrations, UPDRS-III and MoCA scores
						UPDRS-III and MoCA scores	
Mischley et al. (2016)	Dec- 14	Mar-15	15	15	0	GSH measurement by MRS	GSH levels in the brain
Coles et al. (2018)	Sep-14	Sep-15	8 (5 with PD, 3 healthy)	7 (4 with PD and 3 healthy)	1 - withdrew after 5 days due to increased tremor	Age, ethnicity, sex, disease duration, weight, smoking status, use of alcohol and illicit drugs	At 28 days: Blood collected approx 30, 60, 120, 180, 240, 300 and 360 min after final NAC dose
						UPDRS scores (I–III), Hoehn and Yahr staging, medications, supplements, and vital signs	2 h after final NAC dose, brain GSH levels measured using 7T MRS then 3T MRS
						Brain GSH, measured by MRS scans at 3 T and 7 T	
						NAC, Cys, GSH, GSH/GSSG, catalase, MDA and 4-HNE through blood sample collection	
Monti et al. (2016)	Jun-14	Aug-15	23	23	0	DAT binding using SPECT with I-123 Ioflupane	DaTscan and UPDRS
Monti et al. (2019)	Jun-14	Dec-16	42	42	0	DaTscan SPECT imaging and UPDRS scores	DaTscan SPECT imaging and UPDRS scores
Mischley et al. (2015)	Jul-12	Jan-16	30	28	2 withdrew − 1 due to schedule conflicts and 1 due to ringing in her head	NR	Complete blood count (CBC), alanine aminotransferase (ALT), aspartate aminotransferase (AST), blood urea nitrogen (BUN), creatinine, and a urinalysis at 2, 4, 8, 12, 16 weeks. MOSES to assess side effects, SNOT-20 to assess rhinosinusitus, UPDRS, Sensonics Smell ID Test
Mischley et al. (2017)	Apr-15	Apr-16	45	40	5 withdrew − 1 in placebo group due to chronically irritated sinus and headaches	Gender, age, race/ethnicity, years since diagnosis, Hoehn & Yahr stage	PROMIS Global, UPDRS questions 1–17, Non-motor Symptom Score, PDQ-39, and PRO-PD, measures of oxidative stress and defense, H-MRS (15 participants)
					2 in 300 mg/day group: one broke bone and one reported puffiness under eyes		
					2 in 600 mg/day group: one had tachycardia and newly diagnosed cardiomyopathy and one could not attend study visits		
Hauser et al. (2009)	Jul-08	May-09	21	20	1 - discontinued as too far to travel	Age, disease duration, gender, levodopa equivalents, UPDRS, HY, SE, MMSE	(UPDRS, parts I–III), timed walking, tapping, and clinical global impression of change (CGI-C). For patients with motor fluctuations, motor assessments were obtained when subjects were in the ‘‘ON’’ state
						Supine and standing blood pressure, and ECG at baseline and 10 min after intervention	The predefined efficacy outcome measure of greatest interest was the change in UPDRS ADL 1 motor scores from baseline to week 4

### Results of individual studies

#### Intervention

The interventions used were varied in terms of compound (NAC or GSH), dosage, route of administration, and duration. NAC was administered intravenously (IV) at 150 mg/kg (Holmay et al. 2013) and orally in doses ranging from 7 mg/kg to 6000 mg/day (Katz et al. 2015; Coles et al. 2018; Monti et al. 2016, 2019). Two studies combined oral and IV administration (Monti et al. 2016, 2019) over 3 months. GSH was delivered intranasally with a single 200 mg dose (Mischley et al. 2016), and intranasally at 300–600 mg/day (Mischley et al. 2015, 2017), or via IV (Hauser et al. 2009) at 1400 mg three times per week for four weeks.

#### Change in Parkinson’s disease symptoms

The effect of interventions on PD motor and non-motor symptoms produced varying results (Table 4). Monti et al. (2016, 2019) reported significant improvements in UPDRS total scores and DAT binding in caudate and putamen in the NAC, with a mean decrease of 3.25 points (*P* = 0.004) (Monti et al. 2016) and a mean decrease of 4.29 points over three months, compared to an increase of 2.36 in the control group (Monti et al. 2019). These studies also reported significant increases in DAT binding following NAC treatment, with mean differences of 0.15 in the caudate (*p* = 0.014) and 0.12 in the putamen (*p* = 0.039) (Monti et al. 2016) and mean DAT binding increases of 0.092 in the caudate and 0.135 in the putamen (Monti et al. 2019). Mischley et al. (2015, 2017) observed improvements in UPDRS scores with intranasal (in)GSH (–4.6 (4.7), *P* = 0.0025), though the effects were not statistically superior to placebo. Hauser et al. (2009) also found no significant improvement in motor symptoms or ADL UDPRS motor scores between IV GSH and placebo over four weeks (*P* = 0.32) however, this study did report a statistically significant improvement of 10.17 points in non-motor symptom score in 600 mg/d cohort. Notably, Coles et al. (2018) reported worsening of symptoms in 3 of the 5 patients with PD receiving high-dose oral NAC, which resolved after discontinuation.

#### Change in blood/brain GSH levels

The interventions led to varying changes in blood and brain GSH levels. IV NAC significantly increased brain GSH levels, with a maximum change of 55% observed in patients with PD (Holmay et al. 2013). Oral NAC produced a dose-dependent increase in CSF NAC concentrations but did not consistently elevate CSF or brain GSH levels (Katz et al. 2015; Coles et al. 2018). Intranasal GSH administration resulted in a gradual increase in brain GSH levels measured via H-MRS, with mean differences as GSH/Creatine peak ratios ranging from baseline rising from 0.0089 at 7.5 min post dose as midpoint of scan to 0.0201 at 19.9 min, 0.0215 at 32 min, and 0.0340 at 44.7 min (Mischley et al. 2016). However, subsequent studies reported no significant differences in systemic oxidative stress biomarkers or brain GSH levels compared to placebo (Mischley et al. 2017). Moreover, Hauser et al. (2009) did not find significant improvement in GSH biomarkers following IV GSH therapy from baseline to week 4.

### Effect sizes

The UPDRS is a cumulative severity scale in which higher scores represent greater motor and non-motor impairment, therefore, reductions in total or subscale scores reflect clinical improvement, while increases indicate worsening of symptoms. Effect size calculations for UPDRS scores pre- and post-NAC treatment indicated large effects in Monti et al. (2016) (d = 1.07) and Monti et al. (2019) (d = 1.17), demonstrating significant UPDRS score reductions and increased DAT binding. Intranasal GSH in Mischley et al. (2015) and Mischley et al. (2017) also showed large effects (d = 1.08 and d = 0.94), though improvements were not statistically superior to placebo. Hauser et al. (2009) reported a moderate effect (d = 0.53) for IV GSH with no significant symptom improvement. In contrast, Coles et al. (2018) reported symptom worsening with high-dose NAC, yielding a negative effect size (d = -0.87). Katz et al. (2015) stated no significant change in UPDRS scores pre- and post-NAC, but the exact scores were not available in the paper; therefore, the effect size was not calculated for this study.

### MCID changes

The clinical significance of reported changes in UPDRS scores should be considered with comparison to established minimal clinically important differences (MCIDs); defined as a threshold between 4 and 8 points, derived from UPDRS total scores and between 3 and 5 points, derived from UPDRS part III motor scores (Mishra et al. 2024). Four studies demonstrated improvements that met or exceeded the lower bound for clinical relevance. Monti et al. (2016) and Monti et al. (2019) both reported reductions of − 4.3 points, representing minimal but clinically meaningful motor improvements. Larger effects were observed in Mischley et al. (2015) with a total score reduction of − 6.5 points and Mischley et al. (2017) with a reduction of–5.4 points, both within the MCID range and thus considered clinically relevant. However, improvements in symptoms in the study by Mischley et al. (2017) were not shown to be statistically significant from the placebo group. By contrast, Hauser et al. (2009) reported a − 2.8 point change, which did not reach the MCID threshold and was therefore unlikely to reflect a meaningful benefit in motor symptoms for patients. Notably, Coles et al. (2018) demonstrated a + 4.0 point worsening in total UDPRS score, indicating a clinically significant deterioration. Overall, these findings suggest that while several interventions produced improvements of clinical importance, effects were inconsistent across studies. Therefore, larger, double blind RCTs are needed to determine whether NAC or GSH treatment could improve clinical PD symptoms.

### Safety and adverse effects

NAC and GSH were mostly well-tolerated in the included studies, with a low incidence of serious adverse effects. Four studies were completed with no withdrawals. Katz et al. (2015) had one withdrawal due to a post-lumbar puncture headache. Coles et al. (2018) reported three patients had a mild to moderate increase in tremor, one of whom led to discontinuation from the study. One participant in this study developed freezing of gait, but symptoms returned to baseline within two weeks after discontinuation of NAC. Mischley et al. (2015) had one withdrawal due to schedule conflicts and one due to ringing in their head. Mischley et al. (2017) had five withdrawals in total. One of these was from the placebo group due to headaches and irritated sinuses, two were from the 300 mg/day group due to a broken bone and the other due to puffiness under the eyes, and two were from the 600 mg/day group, one due to newly developed cardiomyopathy and tachycardia, and the other not being able to attend study visits. Hauser et al. (2009) had one withdrawal due to the far distance to travel to the study. Minor side effects were also observed, such as gastrointestinal symptoms including indigestion, flatulence, nausea and diarrhoea. Other reported side effects included headache, fatigue, muscle soreness, laboured breathing, sore throat, and increased thirst.

### Risk of bias within studies

All the randomised controlled trials were assessed using the Cochrane risk of bias tool to determine the overall quality of each study (Table 5). Of these 5 studies, 3 were rated as low risk of bias (Mischley et al. 2015, 2017; Hauser et al. 2009), demonstrating adequate randomisation, allocation concealment, and handling of incomplete outcome data. The remaining 2 studies had an unclear overall risk of bias; Monti et al. (2016, 2019) due to high risk in participant and outcome assessor blinding, which, combined with small sample sizes, may have amplified performance and detection biases.

Non-randomised studies were assessed for bias using the Newcastle–Ottawa Scale (Table 6). Of these 4 studies, 2 were rated as low risk of bias (Holmay et al. 2013; Katz et al. 2015), and the remaining 2 studies had a moderate risk of bias (Mischley et al. 2016; Coles et al. 2018) due to limited control for confounders and outcome assessment. Across all of the studies, small sample sizes, short follow-up times and methodological limitations may affect the robustness and generalisability of outcomes.

**Table 4:** Interventions used and study results

References	Intervention/exposure	Dosage	Route of administration	Duration of intervention	Control group	Change in PD motor symptoms	Change in PD non-motor symptoms	Change in blood/brain GSH levels & other biomarkers	Conclusion
Holmay et al. (2013)	150 mg/kg NAC infusion	150 mg/kg	IV	2 h	Healthy controls	N/A	N/A	Blood GSH/GSSG increased following start of NAC transfusion, peaking at 60–75 min. Brain GSH increased peaking at 90–110 min after start of infusion. Max % change in brain GSH 55% (PD), 41% (GD), 34% (HC)	Brain GSH and blood GSH redox ratios increase following IV NAC administration
Katz et al. (2015)	Oral NAC dosage in solution form of either 7 mg/kg, 35 mg/kg, or 70 mg/kg twice daily, or capsule form 70 mg/kg	7 mg/kg, 35 mg/kg or 70 mg/kg	Oral	2 days	Comparison to baseline	No significant changes in MoCA (*p* = 0.29) or UPDRS-III (*p* = 0.86). Scores post intervention, and therefore mean differences, not reported.	N/A	Dose-dependent increase in CSF reduced NAC and total NAC. Highest dose produced concentration of 9.26 No consistent change in CSF cysteine or GSH concentration	Orally administered NAC crosses BBB and produces dose dependent increase in CSF NAC
Mischley et al. (2016)	1 cm3 saline containing 200 mg GSH.	200 mg	Intranasal	1 h	Comparison to baseline	N/A	N/A	Mean difference in brain GSH from baseline: 7.5 min − 0.00890 19.9 min − 0.0201 32.0 min − 0.0215 44.7 min − 0.0340	Intranasal GSH can increase CNS GSH levels
Coles et al. (2018)	Two 3000 mg doses of NAC per day (6000 mg/day), one in morning and one in evening.	2 × 3000 mg (6000 mg/day)	Oral	4 weeks	Healthy controls	Patients with PD: UPDRS scores 20–42 (mean = 32.6) pre-NAC to 24–48 (mean = 36.6) post-NAC Mean difference : -4	N/A	GSH/GSSG ratio from 3.1 to 13.1 pre-NAC to 5.87 to 26.9 post-NAC - significant increase (rank test)	Increased Cys, GSH/GSSH and catalase. No increase in GSH brain concentration. Some people had worsening of PD symptoms, resolved after discontinuation of therapy
						Healthy controls: UPDRS scores 3–4 (mean = 3.6) pre-NAC to 4–7 (mean = 5.6) post-NAC Mean difference: -2		No statistically significant effect on brain GSH- ~6% change with 7T, ~ 10% change with 3T. NAC and Cys concentrations significantly elevated from baseline, plasma GSH almost unchanged	
Monti et al. (2016)	600 mg NAC tablets 2 times a day on days not receiving IV NAC (50 mg/kg mixed into 200 ml of D5W infused over ~1 h once a week	50 mg/kg mixed into 200 ml of D5W + 600 mg NAC tablets 2x a day on days not receiving IV NAC	Oral and IV	3 months	Waitlist control	Significant decrease in UPDRS scores from pre to post-treatment in NAC group (mean difference − 3.25, *p* = 0.004) No significant change in control group pre to post treatment (mean difference − 2.00, *p* = 0.069)	N/A	Significant increase in DAT binding from pre treatment compared to post treatment. (mean difference: 0.15 caudate *p* = 0.014, 0.12 putamen *p* = 0.039). significant difference for caudate (mean difference: -0.09 *p* = 0.171)and significantly negative for putamen(mean difference: -0.14 *p* = 0.027)	NAC might positively impact dopamine function and potentially clinical symptoms
						No significant change in treatment group compared to control (mean difference -1.25, *p* = 0.397)		Changes were significantly higher in treatment group compared to controls for caudate and putamen. (caudate mean change 0.23 *p* = 0.007, putamen mean change 0.26 *p* = 0.003). Significant change in midbrain serotonin transporter binding in NAC group	
						Significant correlation between change in UPDRS scores and change in DAT binding in caudate and putamen			
Monti et al. (2019)	Oral NAC 600 mg twice a day with meals, IV NAC – 50 mg/kg mixed into 200 mL of dextrose 5% in water - infused for ~1 h once a week on days they did not take NAC tablets	50 mg/kg n 200 mL dextrose in water + 600 mg twice a day on other days	Oral and IV	90 days	Waitlist control	UPDRS motor scores significantly improved in NAC group (mean 2.88 decrease), total UPDRS mean decrease of 4.29, compared 2.36 increase in control	UPDRS non-motor scores significantly improved in NAC group (mean 1.41 decrease)	Changes in caudate or putamen were significantly higher compared to control group in NAC group NAC: mean caudate change in DAT binding − 0.092 NAC: mean putamen change in DAT binding − 0.135 Control: mean caudate change in DAT binding - -0.059 Control: mean putamen change in DAT binding - -0.110	Combination of oral and IV NAC improved symptoms UPDRS and increased DAT binding
Mischley et al. (2015)	GSH 600 mg/day, GSH 300 mg/day or placebo − 1 mg syringe 3 times a day for 3 months	600 mg/day, 300 mg/day or placebo	Intranasal	3 months	Placebo	UPDRS scores improved over placebo-total UPDRS mean decrease from baseline: placebo: -1.1 300 mg/d: -5.3 600 mg/d: -4.3	N/A	No statistically significant differences in the frequency of laboratory events as defined by CBC, WBC with differential, ALT, AST, creatinine, blood urea nitrogen, uric acid or urinalysis.	This study supports safety and tolerability of (in)GSH in patients within 10 years of PD diagnosis
Mischley et al. (2017)	200 mg GSH/ml, 100 mgGSH/ml or placebo three times a day for 12 weeks, with 4 week washout period	100 mg GSH/ml or 200 mg GSH/ml 3 times a day	Intranasal	12 weeks	Placebo - saline	Improvement in UPDRS score, but treatment group not superior to placebo UPDRS mean changes (SD) from baseline to 12 weeks: Placebo: -3.4 (6.2) 300 mg/d: -2.6 (7.4) 600 mg/d: -4.6 (4.7)	Statistically significant improvement in non-motor symptom score in 600 mg/d cohort (mean 10.17 point improvement)	No statistical significant between biological markers from baseline and between groups - blood cysteine: sulfate ratios, blood cysteine: cystine ratios, blood GSH peroxidase, blood superoxide dismutase (SOD), blood lipid peroxides, urine lipid peroxides, and urine 8-OHdG Placebo and 600 mg/d treatment groups had a statistically significant decrease in whole blood GSH. No statistically significant increase in brains GSH measured by H-MRS	(in)GSH is not superior to placebo after a 3 month intervention
Hauser et al. (2009)	1400 mg GSH or placebo diluted in 10 mL of normal saline on Monday, Wednesday and Friday over 4 week period	1400 mg glutathione GSH diluted in 10 mL saline	IV	4 weeks	Placebo	No significant difference between groups in UPDRS ADL + motor scores from baseline to week 4 GSH group from 37.6 to 34.8 (mean difference 2.8) Placebo stayed same at 35.1 (mean difference 0)	N/A	N/A	No significant improvement in PD symptoms compared to placebo group. No withdrawals because of adverse effects
						No significant difference from weeks 4–12 GSH group 34.8 to 36.3 (mean difference − 1.5) Placebo 35.1 to 33.1 (mean difference 2)			

**Table 5:** Cochrane risk of bias tool ratings of included studies

Study	Random sequence generation	Allocation concealment	Blinding of participant/personnel	Blinding of outcome assessment	Incomplete outcome data	Selective reporting	Overall
Monti et al. (2016)	+	+	-	-	+	+	?
Monti et al. (2019)_	+	+	-	-	+	+	?
Mischley et al. (2015)	+	?	+	?	+	+	+
Mischley et al. (2017)	?	?	+	+	+	+	+
Hauser et al. (2009)	+	+	+	?	+	+	+

*+*, low risk of bias; –, high risk of bias; ? unclear risk of bias

**Table 6:** Quality assessment of included studies based on the Newcastle-Ottawa scale

Selection					Comparability		Outcome		
Study ID	Representative of the sample	Sample size	Non-respondents	Ascertainment of the exposure (risk factor)	Subjects in different outcome groups are comparable based on study design or analysis	Confounding factors are controlled	Assessment of outcome	Statistical test	Total
Holmay et al. (2013)	*	*	*	*	*	*	*		7
Katz et al. (2015)	*	*	*	*	*		*	*	7
Mischley et al. (2016)	*	*	*	*	*			*	6
Coles et al. (2018)	*		*	*	*	*		*	6

* indicates that the study met the methodological criterion and was awarded one star. A maximum of 4 stars can be awarded for selection, 2 for comparability, and 3 for outcome domains, giving a maximum total of 9 stars

Bias rating: Low risk of bias = 7+, Medium risk of bias = 3–6, High risk of bias = 0–2

## Discussion

This systematic review aimed to investigate the potential role of NAC and GSH as a therapeutic intervention for PD, with encouraging results related to oxidative stress modulation and clinical outcomes. The results suggest mixed findings for the primary outcome of change in UPDRS score. While four studies showed large effect sizes and UPDRS scores indicating improvement in PD symptoms, only three of these were statistically significant. The remaining three studies reported no significant improvements in UPDRS scores. There was no indication that NAC or GSH had a different effect on PD symptoms. Therefore, the findings remain inconsistent on whether UPDRS scores can be improved with NAC or GSH treatment. This may be due to the wide variability in study designs, and heterogeneity in presentation of PD symptoms, which can greatly vary between patients (Greenland et al. 2019).

Two studies (Holmay et al. 2013; Coles et al. 2018) demonstrated a significant increase in the GSH/GSSG ratio in the brain of patients with PD after NAC administration, indicating enhanced redox balance and reduced oxidative stress. These findings align with the established mechanism of NAC as a precursor to GSH, the brain’s primary antioxidant, which helps combat ROS and oxidative damage, a critical factor in PD pathophysiology. Katz et al. (2015) reported a dose-dependent increase in NAC levels in the CSF, supporting systemic absorption and CNS-proximal exposure; however, CSF concentrations may not directly reflect brain parenchymal levels, and transport across the blood–CSF barrier should be considered. Two studies also reported increased brain GSH or NAC levels (Holmay et al. 2013; Monti et al. 2016), supporting the hypothesis that NAC restores antioxidant capacity within the brain. In addition to biochemical changes, NAC demonstrated clinically relevant benefits. For example, Monti et al. (2016, 2019) observed a significant improvement in UPDRS scores and a decrease in both motor and non-motor symptoms in NAC-treated groups compared to controls. This improvement was accompanied by an increase in DAT binding on DaTscan imaging, suggesting a potential neuroprotective effect and enhancement of dopaminergic function. While NAC demonstrated promising clinical and mechanistic effects at moderate dosing regimens, evidence from higher-dose administration raises important safety considerations. Coles et al. (2018) reported clinically meaningful worsening of Parkinson’s disease symptoms in patients receiving high-dose oral NAC, with symptom resolution following treatment discontinuation, suggesting a potential dose-dependent adverse effect. In addition, one trial reported withdrawal due to tachycardia and newly diagnosed cardiomyopathy in a participant receiving GSH dosage; although causality could not be established, symptoms resolved after treatment cessation and experimental research in mice suggest that excessive antioxidant exposure may induce reductive stress and cardiomyocyte injury (Mischley et al. 2017). Collectively, these findings underscore the need for cautious dose optimisation, cardiovascular monitoring, and avoidance of empirical antioxidant dose escalation outside of controlled clinical trials with rigorous safety monitoring.

NAC is understood to be generally safe and well tolerated as it is a commonly used drug and is FDA approved for 72 h oral or 21 h IV doses following paracetamol overdose (Slattery et al. 2015). In a review of NAC usage in psychiatric and neurologic disorders, the most common dosages were 2000–2400 mg/day, which were generally well tolerated with low incidence of serious adverse effects (Slattery et al. 2015). Different methods of administration could potentially affect the bioavailability of the drug, for example, Coles et al. (2018) found that there may be limited CNS penetration in oral administration compared to IV, as shown by increases in GSH/GSSG and catalase but not cerebral GSH levels. IV administration may also not be sustainable for the long term (Tenório et al. 2021). The ability of NAC to cross the BBB has been unclear, but could depend on its dosage and administration, as different methods could result in different mechanisms of crossing the blood vessel wall. The BBB is formed by specialised endothelial cells connected by tight junctions, supported by astrocytic end-feet and pericytes, which together regulate molecular transport into the brain parenchyma. Given its small molecular size and hydrophilic properties, NAC may cross the BBB to a limited extent via passive diffusion; however, transport is also likely influenced by carrier-mediated systems and concentration gradients (Bavarsad Shahripour et al. 2014). Further studies are needed to determine the most ideal dosage and administration route for a greater bioavailability of NAC. Trials using NAC have been carried out lasting for up to 60 months in psychiatric disorders, but there is limited research on long-term trials in PD and whether NAC could be used for an extended period in PD remains unclear. Further research with long-term follow-up, a larger sample size, and more diverse participant characteristics is necessary to ensure the safety of NAC or GSH as interventions in PD.

The role of GSH as a direct therapeutic intervention in PD showed varying results in the included studies. In Mischley et al. (2016), intranasal administration of GSH led to slight increases in brain GSH levels as measured by H-MRS. The study reported incremental increases in brain GSH over time, peaking at approximately 44 min post-intervention. This finding suggests that intranasal GSH may have some capacity to cross the BBB and increase CNS GSH levels; however, intranasal delivery can also reach the CNS via olfactory or trigeminal pathways, bypassing the BBB (Hanson and Frey 2008). Mischley et al. (2017) extended this investigation by evaluating the effects of intranasal GSH on oxidative stress markers and PD symptoms over a 12-week intervention period. While there were reported improvements in non-motor symptom scores for the 600 mg/day treatment group, no statistically significant changes in motor symptoms were observed when compared to placebo. These findings were also reported by Hauser et al. (2009) who found no significant difference between groups in changes of motor scores after four weeks of GSH administration.

The variability in GSH findings can be attributed to differences in delivery methods and bioavailability. Intranasal administration of GSH may provide better CNS penetration than systemic route, however, the extent of brain uptake and its translation into meaningful clinical effects remains unclear. GSH has been reported to break down rapidly in the body, which means it may not sufficiently be able to cross the BBB (Tardiolo et al. 2018). Intranasal administration can provide a direct nose-to-brain pathway via olfactory and trigeminal nerve routes, thereby reducing first-pass systemic degradation and potentially enhancing CNS exposure. However, even with this alternative route, the extent of brain uptake and its translation into meaningful clinical effects remains unclear. Additionally, the short duration of one hour for the administration of GSH in the study by (Mischley et al. 2016) to 12 weeks (Mischley et al. 2017) may have limited the detection of sustained changes in disease progression. In this review, there was a low incidence of serious adverse effects following NAC and GSH treatment, but all of the included studies had a small sample size and were carried out in the United States.

### Limitations of the study

This systematic review assessed studies that utilised GSH and its precursor, NAC, to evaluate the therapeutic efficacy in treating symptoms of PD. All reviewed articles comprised of both randomised and non-randomised intervention studies. However, some limitations were identified across the included studies, which may have affected the interpretation of these findings. The small number of included studies and limited sample sizes (Holmay et al. 2013; Coles et al. 2018) may have limited the detection of significant changes in motor and non-motor symptoms or oxidative stress markers. Although, short durations of intervention, Holmay et al. (2013) and Katz et al. (2015) reported effects of NAC administration over 2 h to 2 days, whereas longer-term data over 3 months are needed to assess sustained neuroprotective effects and clinical benefits (Monti et al. 2019). A lack of standardisation of trials in terms of dosage, route of administration, and outcome measures was observed across the studies. Restriction to English-language publications and the fact that all included studies were conducted in the United States limit the external validity of the results due to lack of ethnic diversity, differences in healthcare systems, and geographic generalisability. In addition, assessing both RCTs and non-randomised studies meant there were large differences between studies included in the review. Combined with the clinical heterogeneity of PD with how symptoms present, the included studies did not meet the minimum requirements for quantitative synthesis such as comparable populations, intervention regimens, outcome definitions, and effect measures with consistently reported variance hence, meta-analysis was not performed. Four out of the nine included studies had an unclear or moderate risk of bias, which may have impacted the reliability of the results. NAC dosages varied across studies, ranging from 7 mg/day (Katz et al. 2015) to 6000 mg/day (Coles et al. 2018). Moreover, oral or IV routes of administration make direct comparisons more challenging.

### Recommendations for future research

Future research studies should prioritise adequately powered, multicentre, double-blind RCTs of NAC in PD with rigorous controls, standardised dosing regimens, clearly defined routes of administration, and stratification by disease stage and baseline oxidative stress markers to identify patient subgroups most likely to benefit. Priority should also be given to studies that apply standardised outcome measures, including UPDRS with MCID reporting, DAT imaging, and validated oxidative stress biomarkers. Longitudinal studies with sufficient durations of at least 12–24 months are essential to assess sustained neuroprotective benefits and potential disease-modifying impact. Methodological innovations such as standardised pharmacokinetic profiling, stratified analyses based on bioavailability, and the use of CONSORT and PRISMA guidline reporting would improve comparability and reproducibility. Establishing consensus on core outcome sets, minimum reporting standards, and shared dosing frameworks would substantially strengthen the evidence base, reduce heterogeneity and enable future meta-analyses.

## Conclusion

This systematic review revealed NAC as a promising intervention compound to alleviate oxidative stress and improve clinical outcomes in PD. Evidence supports its ability to increase GSH levels, enhance dopamine function, and improve motor and non-motor symptoms. However, the efficacy of GSH for the treatment of PD remains unclear due to conflicting findings. Furthermore, given the limited and heterogeneous evidence base, together with dose-dependent adverse effects and reported cardiac safety signals, high-dose NAC or GSH should not be used routinely as disease-modifying therapies in PD outside clinical trials. Larger, longer-term RCTs with standardised methodologies, careful dose optimisation, systematic cardiovascular monitoring, and long-term follow-ups are needed to validate these preliminary findings and advance the long-term efficacy of NAC and GSH in PD management.

## Supplementary Information

Below is the link to the electronic supplementary material.

Supplementary Material 1

## Data Availability

Data sharing does not apply to this article as no new data were created.

## Abbreviations

PD: Parkinson’s disease
NAC: N-acetylcysteine
GSH: Glutathione
GSSG: Glutathione disulfide
UPDRS: Unified Parkinson’s Disease Rating Scale
CSF: Cerebrospinal fluid
DAT: Dopamine transporter
MRS: Magnetic resonance spectroscopy
MPP: 1-methyl-4-phenylpyridinium
MPTP: 1-methyl-4-phenyl-1,2,3,6-tetrahydropyridine
ADL: Activities of daily living
MoCA: Montreal cognitive assessment
4-HNE: 4-hydroxynonenal
6-OHDA: 6-hydroxydopamine
L-DOPA: Levodopa
ECG: Electrocardiogram
SPECT: Single-photon emission computed tomography
PROMIS: Patient-reported outcomes measurement information system
PDQ-39: Parkinson’s Disease Questionnaire-39
PRO-PD: Patient-reported outcomes in Parkinson’s disease
H&Y: Hoehn and Yahr
MMSE: Mini-mental state examination
CBC: Complete blood count
ALT: Alanine aminotransferase
AST: Aspartate aminotransferase
BUN: Blood urea nitrogen
SOD: Superoxide dismutase
MCID: Minimal clinically important difference
SD: Standard deviation
