# Supplementary material for: The role of N-acetylcysteine and glutathione in the management of Parkinson’s disease: a systematic review of oxidative biomarkers and clinical outcomes
Source: Amino Acids. 2026 Mar 24;58:26. doi: 10.1007/s00726-026-03513-5 (PMC13050330; doi:10.1007/s00726-026-03513-5)
Supplement: Supplementary file 1 [file 726_2026_3513_MOESM1_ESM.docx]

**Supplementary Material**

**S1. Search term coding for different databases**

| Database | Search Terms Formular | Filter applied |
| --- | --- | --- |
| Cochrane | Title, Abstract, Keyword  (Parkinson's disease OR Parkinsonism OR Parkinsonian symptoms) AND (N-acetylcysteine OR NAC OR N-acetyl-L-cysteine OR N Acetyl Lcysteine OR N Acetylcysteine OR acetylcysteine OR glutathione OR GSH OR GSSG) | Date:  2003-2024 |
| Ovid | Title  (Parkinson's disease OR Parkinsonism OR Parkinsonian symptoms) AND (N-acetylcysteine OR NAC OR N-acetyl-L-cysteine OR N Acetyl Lcysteine OR N Acetylcysteine OR acetylcysteine OR glutathione OR GSH OR GSSG) | Date:  2003-2024 |
| ProQuest | TI,AB("Parkinson's disease" OR "Parkinsonism" OR "Parkinsonian symptoms") AND TI,AB("N-acetylcysteine" OR "NAC" OR "N-acetyl-L-cysteine" OR "N Acetyl Lcysteine" OR "N Acetylcysteine" OR "acetylcysteine" OR "glutathione" OR "GSH" OR "GSSG") | Date:  2003-01-01 to 2024-12-31  Language:  English  Full-text |
| PubMed | (Parkinson's disease[Title] OR Parkinsonism[Title] OR Parkinsonian symptoms[Title]) AND (N-acetylcysteine[Title] OR NAC[Title] OR N-acetyl-L-cysteine[Title] OR N Acetyl Lcysteine[Title] OR N Acetylcysteine[Title] OR acetylcysteine[Title] OR glutathione[Title] OR GSH[Title] OR GSSG[Title]) | Date:  2003-2024 |
| Scopus | Title, Abstract, Keyword  (Parkinson's disease OR Parkinsonism OR Parkinsonian symptoms) AND (N-acetylcysteine OR NAC OR N-acetyl-L-cysteine OR N Acetyl Lcysteine OR N Acetylcysteine OR acetylcysteine OR glutathione OR GSH OR GSSG) | Date:  2003-2024  Language:  English |
| Web of Science | Title, Abstract, Keyword  (Parkinson's disease OR Parkinsonism OR Parkinsonian symptoms) AND (N-acetylcysteine OR NAC OR N-acetyl-L-cysteine OR N Acetyl Lcysteine OR N Acetylcysteine OR acetylcysteine OR glutathione OR GSH OR GSSG) | Date:  2003-2024 |
